# Misfolding-induced chronic pancreatitis in *CPA1 N256K* mutant mice is unaffected by global deletion of *Ddit3/Chop*

**DOI:** 10.1038/s41598-022-09595-x

**Published:** 2022-04-15

**Authors:** Balázs Csaba Németh, Alexandra Demcsák, Andrea Geisz, Miklós Sahin-Tóth

**Affiliations:** 1 Department of Surgery, University of California Los Angeles, Los Angeles, CA 90095 USA; 2 Department of Molecular and Cell Biology, Boston University Henry M. Goldman School of Dental Medicine, Boston, MA 02118 USA

**Keywords:** Acute pancreatitis, Chronic pancreatitis

## Abstract

Genetic mutations in pancreatic digestive enzymes may cause protein misfolding, endoplasmic reticulum (ER) stress and chronic pancreatitis. The *CPA1 N256K* mouse model carries the human p.N256K carboxypeptidase A1 (CPA1) mutation, a classic example of a pancreatitis-associated misfolding variant. *CPA1 N256K* mice develop spontaneous, progressive chronic pancreatitis with moderate acinar atrophy, acinar-to-ductal metaplasia, fibrosis, and macrophage infiltration. Upregulation of the ER-stress associated pro-apoptotic transcription factor *Ddit3/Chop* mRNA was observed in the pancreas of *CPA1 N256K* mice suggesting that acinar cell death might be mediated through this mechanism. Here, we crossed the *CPA1 N256K* strain with mice containing a global deletion of the *Ddit3/Chop* gene (*Ddit3-KO* mice) and evaluated the effect of DDIT3/CHOP deficiency on the course of chronic pancreatitis. Surprisingly, *CPA1 N256K* x *Ddit3-KO* mice developed chronic pancreatitis with a similar time course and features as the *CPA1 N256K* parent strain. In contrast, *Ddit3-KO* mice showed no pancreas pathology. The observations indicate that DDIT3/CHOP plays no significant role in the development of misfolding-induced chronic pancreatitis in *CPA1 N256K* mice and this transcription factor is not a viable target for therapeutic intervention in this disease.

## Introduction

The exocrine pancreas produces and secretes a variety of digestive enzymes in large quantities. Rarely, genetic mutations can affect the proper folding of these enzymes in the endoplasmic reticulum (ER), which results in so-called “misfolding”^1^. The consequence of misfolding is decreased enzyme secretion from the acinar cells and accumulation and/or degradation of the misfolded protein inside the cells. Misfolding elicits ER stress, which triggers signaling pathways designed to limit the harmful consequences of misfolding and restore the normal folding function of the ER^2–5^. Unresolved ER stress of the exocrine pancreas causes chronic pancreatitis, a progressive inflammatory disorder leading to permanent morphological and functional impairment^1^. Mutations associated with misfolding, ER stress, and chronic pancreatitis have been described in the serine protease 1 (*PRSS1*) gene that encodes human cationic trypsinogen^6–8^, the carboxypeptidase A1 (*CPA1*) gene^9,10^, the carboxyl ester lipase (*CEL*) gene^11–16^, the pancreatic lipase (*PNLIP*) gene^17–21^, and the chymotrypsin C (*CTRC*) gene^22,23^. With the exception of CTRC, these digestive enzymes represent the most abundantly expressed secretory proteins of the pancreas^24^. Heterozygous misfolding variants in *PRSS1*, *CPA1,* and *CTRC* are associated with hereditary or sporadic idiopathic chronic pancreatitis. Heterozygous single-nucleotide deletions in *CEL* cause pancreatic insufficiency and the monogenic diabetes syndrome MODY8, whereas *CEL-HYB1*, a hybrid gene between *CEL* and its neighboring pseudogene increases risk for idiopathic chronic pancreatitis. Homozygous or trans-heterozygous misfolding *PNLIP* variants were reported in rare cases of inborn lipase deficiency^17–19^; whereas association of heterozygous misfolding *PNLIP* variants with chronic pancreatitis has remained uncertain^20,21^.

The best characterized examples of misfolding digestive enzymes are the pancreatitis-associated CPA1 variants. In cell culture experiments, pathogenic mutants such as p.V251M, p.N256K, and p.S282P exhibit an essentially complete secretion defect, intracellular retention of CPA1 and elevation of ER stress markers such as the mRNA for *HSPA5*, encoding a master chaperone called the Binding Immunoglobulin Protein (BiP), spliced *XBP1* encoding the transcription factor X-box-binding protein 1, and *DDIT3* encoding the transcription factor C/EBP Homologous Protein (CHOP)^9,10^. Introduction of the p.N256K mutation into the mouse CPA1 reproduces the human cellular phenotype^25^. Encouraged by this observation, we generated a mouse model which harbors the human p.N256K mutation in the endogenous mouse *Cpa1* gene^25^. Although human carriers are heterozygous, the *CPA1 N256K* mice were bred to homozygosity to obtain a stronger phenotype. Remarkably, *CPA1 N256K* mice develop spontaneous chronic pancreatitis characterized by acinar atrophy, diffuse fibrosis, regenerative pseudotubular complexes, macrophage infiltration and an increase in plasma amylase activity. The disease phenotype is relatively mild yet progressive over the 12-month time-course studied so far. Ethanol feeding accelerates disease progression^26^. Importantly, the pancreas also shows signs of chronic ER stress, indicated by small elevations in the mRNA levels of *Hspa5/BiP*, and more pronounced increases in the *Ddit3/Chop* transcript levels.

Since the pro-apoptotic transcription factor DDIT3/CHOP has been implicated in ER-stress associated cell death, we hypothesized that loss of pancreatic acinar cells may occur via this mechanism in the *CPA1 N256K* mice. In turn, acinar cell death may drive the fibro-inflammatory process of chronic pancreatitis. Consistent with this notion, a large number of studies reported that DDIT3/CHOP plays a role in various diseases and deletion of DDIT3/CHOP may be protective (reviewed in Ref.^27^). In the present study, we generated a *CPA1 N256K* strain with a global deletion of *Ddit3/Chop*, and studied the effect of DDIT3/CHOP deficiency in the development and course of chronic pancreatitis.

## Materials and methods

All methods were performed in accordance with the relevant guidelines and regulations and were in accordance with the ARRIVE guidelines.

### Accession numbers

NM_007837.4, *Mus musculus* DNA-damage inducible transcript 3 (*Ddit3*), mRNA; NM_025350.4, *Mus musculus* carboxypeptidase A1 (*Cpa1*) mRNA.

### Animals

Homozygous *CPA1 N256K* mice containing the human pancreatitis-associated p.N256K *CPA1* mutation in the mouse *Cpa1* locus were reported previously^25^. These mice are on the C57BL/6N genetic background. Genotyping was performed as described^25^. The *Ddit3/Chop*-deleted mouse strain B6.129S(Cg)-*Ddit3*^tm2.1Dron^/J^28^ was purchased from The Jackson Laboratory (Bar Harbor, Maine). For simplicity, we refer to this strain as *Ddit3*-knockout (*Ddit3-KO*). The *Ddit3-KO* mice were backcrossed with C57BL/6N twice and then bred to homozygosity. To genotype for the *Ddit3-KO* and wild-type *Ddit3* alleles, the following primers were used: Common forward primer 5′-ATG CCC TTA CCT ATC GTG-3′, KO reverse primer 5′-AAC GCC AGG GTT TTC CCA GTC A-3′, and *Ddit3* reverse primer 5′-GCA GGG TCA AGA GTA GTG-3′. The KO reverse primer anneals to a NLS-*lacZ* cassette insert and yields a 320 bp fragment. The *Ddit3* reverse primer generates a 544 bp product. The novel *CPA1 N256K* × *Ddit3-KO* strain was generated by crossing the respective parent strains and breeding both alleles to homozygosity. C57BL/6N mice were obtained from Charles River Laboratories (Wilmington, MA) or produced in our breeding facility from the same stock.

### Study approval

Animal experiments were performed at the University of California Los Angeles with the approval and oversight of the Animal Research Committee, including protocol review and post-approval monitoring. Initial breeding was carried out at Boston University with the approval and oversight of the Institutional Animal Care and Use Committee. The animal care programs at these institutions are managed in full compliance with the US Animal Welfare Act, the United States Department of Agriculture Animal Welfare Regulations, the US Public Health Service Policy on Humane Care and Use of Laboratory Animals and the National Research Council's Guide for the Care and Use of Laboratory Animals. The University of California Los Angeles and Boston University have approved Animal Welfare Assurance statements (A3196-01 and A3316-01, respectively) on file with the US Public Health Service, National Institutes of Health, Office of Laboratory Animal Welfare. Both institutions are accredited by the Association for Assessment and Accreditation of Laboratory Animal Care International (AAALAC).

### Histology

Pancreas tissue was fixed in 10% neutral buffered formalin, paraffin-embedded, sectioned, and stained with hematoxylin–eosin or Masson’s trichrome staining, or analyzed by immunohistochemistry (IHC), as indicated, at the Translational Pathology Core Laboratory of UCLA. IHC staining for F4/80, SOX9, and alpha smooth muscle actin (alpha-SMA) was performed using the following antibodies: rabbit monoclonal anti-F4/80 antibody 1:200 dilution for 1 h (Cell Signaling, catalog number 70076), recombinant rabbit monoclonal anti-SOX9 antibody 1:800 dilution for 1 h (Abcam, catalog number ab185230), and rabbit polyclonal anti-alpha-SMA antibody 1:500 dilution for 1 h (Abcam, catalog number ab5694).

### Plasma amylase and lipase assays

Blood was collected through cardiac puncture and plasma was isolated by centrifugation at 2000*g* for 15 min, at 4 °C. Enzyme activity of amylase in 1 µL blood plasma was then determined with the 2-chloro-p-nitrophenyl-α-d-maltotrioside substrate, as reported previously^29^. Lipase activity in blood plasma (1 µL assayed) was measured with the 1,2-*O*-dilaurylrac-glycero-3-glutaric acid-(6′-methylresorufin)-ester substrate, using the Diazyme Lipase Assay Kit (Diazyme Laboratories, Poway, California, USA, catalog number DZ132A), according to the manufacturer’s instructions. Rates of substrate cleavage were expressed in mOD/min units.

### RNA isolation and reverse transcription PCR

Total RNA was extracted from mouse pancreas using approximately 20 mg freshly isolated tissue with the RNeasy Plus Mini Kit (Qiagen, Valencia, CA). Two μg of RNA was reverse-transcribed using the High Capacity cDNA Reverse Transcription Kit (catalog number 4368814, Thermo Fisher Scientific). Complementary DNA for *Ddit3* was amplified using the following primers, which yield a 286 nt amplicon: *Ddit3* mouse sense primer: 5′-CAC ATC CCA AAG CCC TCG CTC TC-3′ and *Ddit3* mouse antisense primer: 5′-TCA TGC TTG GTG CAG GCT GAC CAT-3′. As a house-keeping gene control, reverse-transcribed mouse 18S RNA was amplified, as reported previously^29^.

### Hydroxyproline assay

The hydroxyproline content of the pancreas was determined by the reaction of oxidized hydroxyproline with 4-(dimethylamino)benzaldehyde (Millipore Sigma, catalog number MAK008), as described previously^25^. Values were normalized to the total protein concentration and expressed in units of ng hydroxyproline per µg protein.

### Pancreatic trypsinogen and chymotrypsinogen content

Pancreas tissue (30–40 mg) was homogenized in 300–400 μL 20 mM Na-HEPES (pH 7.4), and the homogenate was cleared by centrifugation (850*g*, 10 min, 4 °C). Levels of protease zymogens were then measured after maximal activation using trypsin and chymotrypsin specific substrates, as described previously^30^.

### Statistics

Experimental results were graphed as individual data points. Where graphically feasible, the mean and standard deviation were also indicated. Differences of means between the groups were analyzed by one-way ANOVA followed by Tukey’s post-hoc test. P < 0.05 was considered statistically significant.

## Results

### Global deletion of *Ddit3/Chop* in *CPA1 N256K* mice

**Figure 1:**
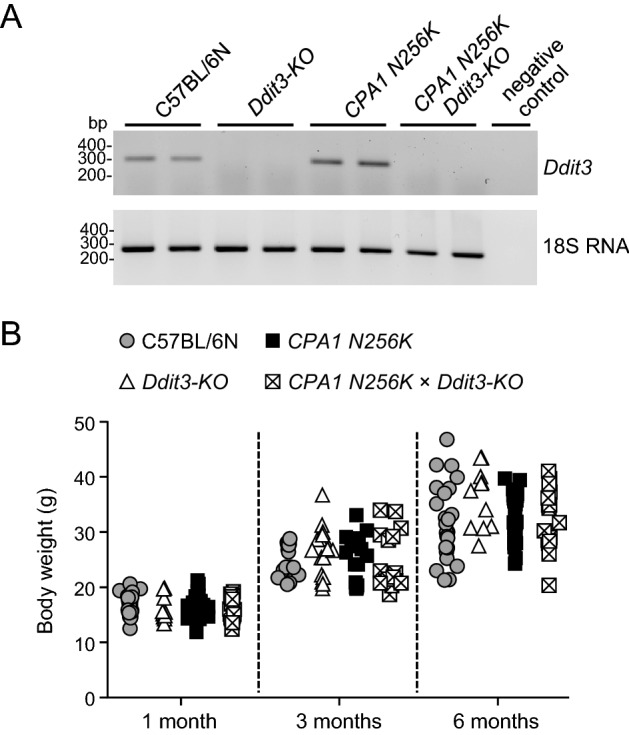
Pancreatic *Ddit3* expression and body weight in *CPA1 N256K* × *Ddit3-KO* mice. (**A**) Reverse-transcription PCR analysis of *Ddit3* mRNA expression in the pancreas of C57BL/6N, *Ddit3-KO*, *CPA1 N256K*, and *CPA1 N256K* × *Ddit3-KO* mice at 6 months of age. A representative agarose gel picture is shown. See “Methods” for details. (**B**) Body weight of mice at 1, 3, and 6 months of age. C57BL/6N (n = 23, 15, and 25), *Ddit3-KO* (n = 10, 17, and 12), *CPA1 N256K* (n = 25, 16, and 25), *CPA1 N256K* x *Ddit3-KO* (n = 18, 16, and 16).

To render the *CPA1 N256K* strain deficient in DDIT3/CHOP, we crossed these mice with a commercially available *Ddit3-KO* strain. Mice were bred to homozygosity for both alleles. RT-PCR analysis of the pancreas for *Ddit3* transcripts confirmed the absence of *Ddit3* expression in *Ddit3-KO* and *CPA1 N256K* × *Ddit3-KO* mice, whereas *Ddit3* was readily detectable in the pancreas of C57BL/6N and *CPA1 N256K* mice (Fig. 1A, Supplementary Fig. 1). As described previously, pancreatic *Ddit3* levels were higher in *CPA1 N256K* mice versus C57BL/6N mice. *CPA1 N256K* × *Ddit3-KO* mice had no discernible phenotype and grew and bred normally. When the body weight of C57BL/6N, *Ddit3-KO*, *CPA1 N256K* and *CPA1 N256K* × *Ddit3-KO* mice was compared at 1, 3, and 6 months of age, no significant differences were observed (Fig. 1B).

### Pancreas atrophy in *CPA1 N256K* × *Ddit3-KO* mice

**Figure 2:**
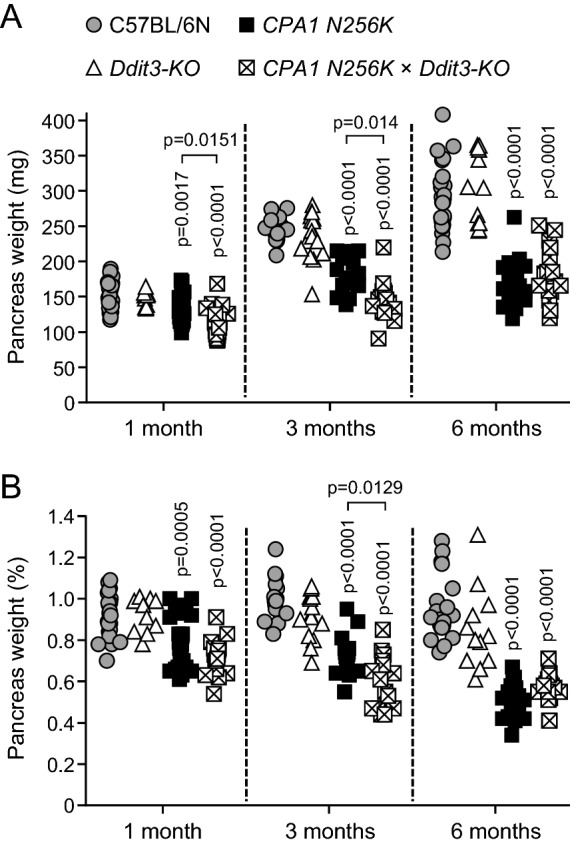
Pancreas weight in *CPA1 N256K* × *Ddit3-KO* mice. (**A**) Pancreas weight of mice at 1, 3, and 6 months of age. C57BL/6N (n = 23, 15, and 25), *Ddit3-KO* (n = 10, 17, and 12), *CPA1 N256K* (n = 25, 16, and 25), *CPA1 N256K* × *Ddit3-KO* (n = 18, 16, and 16). (**B**) Pancreas weight expressed as percent body weight. Individual data points are shown.

One of the easily measurable hallmarks of chronic pancreatitis is the atrophy of the exocrine pancreas which results in reduced pancreatic weight. Assessment of the pancreas weight of C57BL/6N, *Ddit3-KO*, *CPA1 N256K* and *CPA1 N256K* × *Ddit3-KO* mice at 1, 3, and 6 months of age revealed significant atrophy in *CPA1 N256K* and *CPA1 N256K* × *Ddit3-KO* mice relative to the C57BL/6N and *Ddit3-KO* mice (Fig. 2A). There was no difference between the pancreas weight of C57BL/6N and *Ddit3-KO* mice. When *CPA1 N256K* and *CPA1 N256K* × *Ddit3-KO* mice were compared, pancreatic weight loss was slightly more pronounced in *CPA1 N256K* x *Ddit3-KO* at 1 month and 3 months of age. The pancreas atrophy in *CPA1 N256K* and *CPA1 N256K* × *Ddit3-KO* mice remained significant even after normalization of the pancreas weight to body weight (Fig. 2B). The findings indicate that genetic deletion of *Ddit3/Chop* in the *CPA1 N256K* mice does not protect against onset and progression of chronic pancreatitis, as judged by pancreatic atrophy.

**Figure 3:**
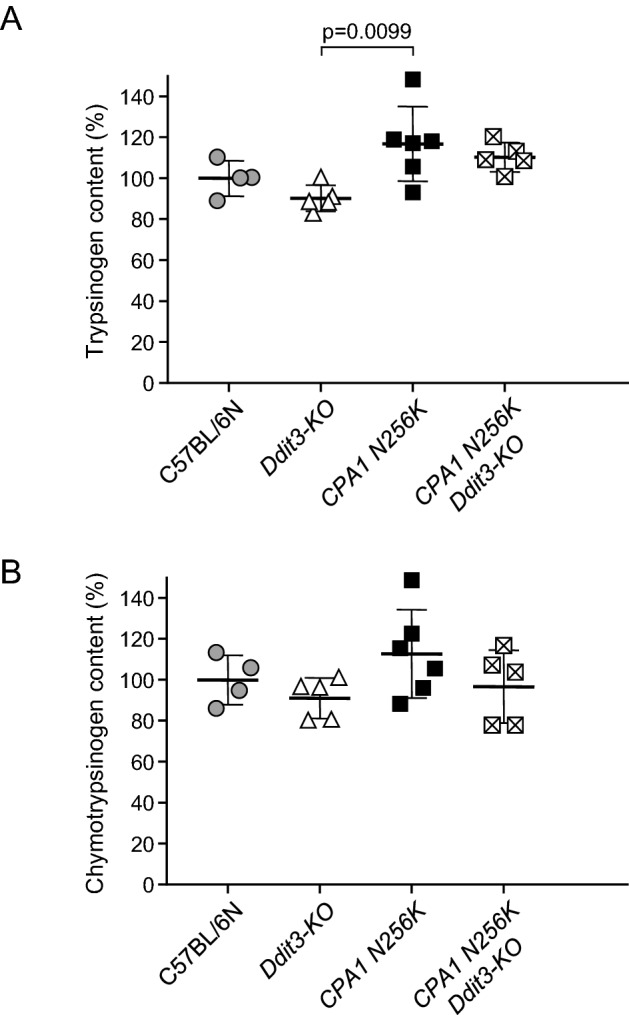
Protease zymogen content of the pancreas from *CPA1 N256K* × *Ddit3-KO* mice. (**A**) Trypsinogen, and (**B**) chymotrypsinogen content was measured from pancreas homogenates of 1-month-old C57BL/6N, *Ddit3-KO*, *CPA1 N256K,* and *CPA1 N256K* × *Ddit3-KO* mice by enzymatic assays after full activation to trypsin and chymotrypsin. Results were expressed as percent of the average C57BL/6N values. Individual data points with mean (horizontal bar) and standard deviation are shown.

To assess whether digestive protease expression might be altered by *Ddit3/Chop* deficiency, we compared the trypsinogen (Fig. 3A) and chymotrypsinogen (Fig. 3B) content in the pancreas of C57BL/6N, *Ddit3-KO*, *CPA1 N256K,* and *CPA1 N256K* × *Ddit3-KO* mice at 1 month of age, when disease activity was only incipient. Interestingly, protease zymogen levels were slightly (by about 10–15%) elevated in *CPA1 N256K* mice relative to C57BL/6N mice, and this trend reached statistical significance for trypsinogen. A non-significant decrease (about 10%) was apparent in *Ddit3-KO* mice, while the *CPA1 N256K* × *Ddit3-KO* cross had normal protease zymogen levels. We consider the observed changes small and biologically not relevant.

### Plasma amylase and lipase in *CPA1 N256K* × *Ddit3-KO* mice

**Figure 4:**
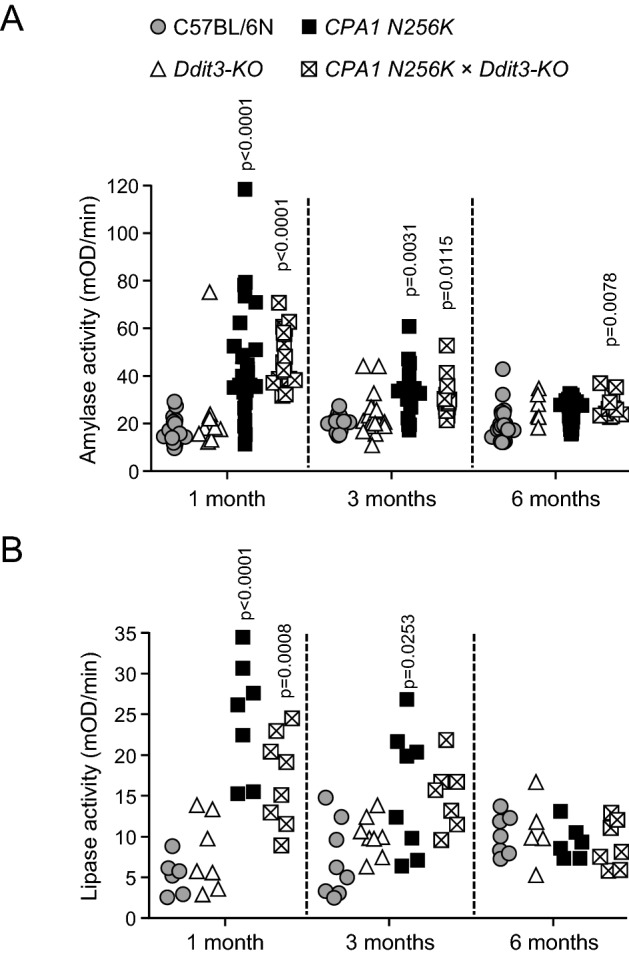
Plasma amylase and lipase activity in *CPA1 N256K* × *Ddit3-KO* mice. (**A**) Plasma amylase activity of mice at 1, 3, and 6 months of age. C57BL/6N (n = 23, 15, and 25), *Ddit3-KO* (n = 10, 17, and 6), *CPA1 N256K* (n = 24, 15, and 11), *CPA1 N256K* × *Ddit3-KO* (n = 18, 16, and 12). (**B**) Plasma lipase activity of mice at 1, 3, and 6 months of age. C57BL/6N (n = 6, 8, and 7), *Ddit3-KO* (n = 7, 8, and 5), *CPA1 N256K* (n = 7, 8, and 6), *CPA1 N256K* × *Ddit3-KO* (n = 8, 7, and 7). Individual data points are shown.

As described previously, there was a significant increase in plasma amylase activity in *CPA1 N256K* mice at 1 month of age, relative to C57BL/6N mice, and the same degree of elevation was observed in *CPA1 N256K* × *Ddit3-KO* mice as well (Fig. 4A). Despite the onset of acinar atrophy, the higher plasma amylase activity in these two strains remained detectable even at 3 months of age. There was no appreciable difference between *CPA1 N256K* and *CPA1 N256K* × *Ddit3-KO* mice, indicating similar severity of pancreatitis. As expected, plasma amylase levels of *Ddit3-KO* mice were comparable to those of C57BL/6N mice. Similar tendencies were apparent when lipase activity was measured on a subset of blood samples from the four mouse strains studied (Fig. 4B).

### Pancreas histology in *CPA1 N256K* × *Ddit3-KO* mice

**Figure 5:**
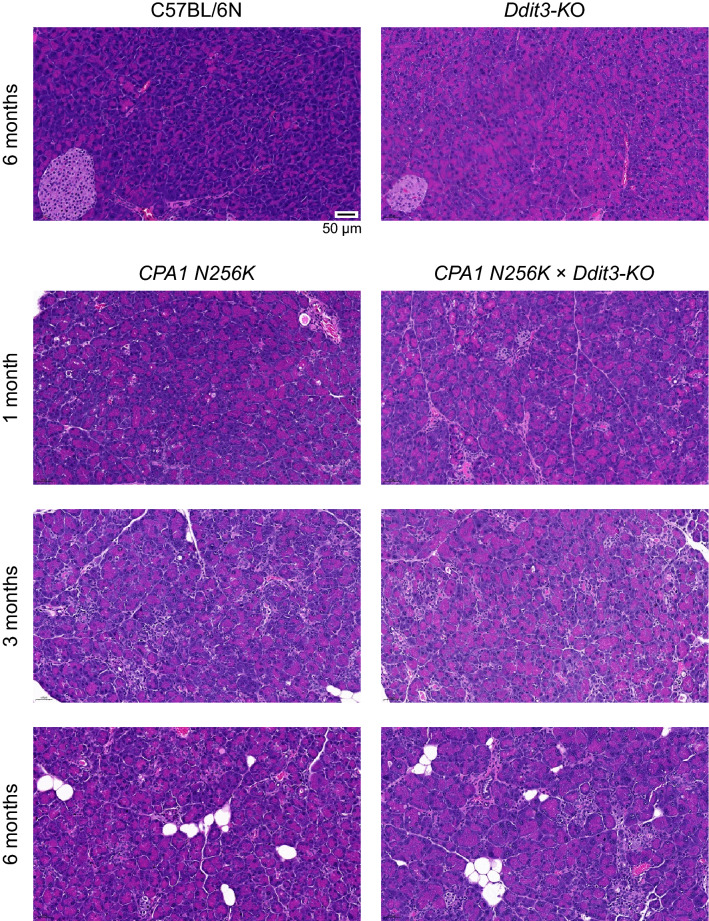
Histology of chronic pancreatitis in *CPA1 N256K* × *Ddit3-KO* mice. Representative hematoxylin–eosin stained pancreas sections are shown from C57BL/6N and *Ddit3-KO* mice at 6 months of age and from *CPA1 N256K* and *CPA1 N256K* × *Ddit3-KO* mice at 1, 3, and 6 months of age. The scale bar corresponds to 50 µm.

To evaluate the histological progression of chronic pancreatitis in *CPA1 N256K* and *CPA1 N256K* × *Ddit3-KO* mice, pancreas sections were stained with hematoxylin–eosin at 1, 3, and 6 months of age (Fig. 5) and compared to similarly treated pancreas sections from C57BL/6N and *Ddit3-KO* mice. Predictably, pancreas histology was completely normal for C57BL/6N and *Ddit3-KO* mice at 6 months of age. In contrast, the previously described progressive histological changes were evident in *CPA1 N256K* and *CPA1 N256K* × *Ddit3-KO* mice. These included scattered acinar cell dropouts and appearance of pseudotubular complexes, with consequent disorganization of the normally tightly packed acinar cell compartment. Relative to 1 month, significantly more histological lesions were apparent at 3 months of age, whereas by 6 months of age fatty replacement was also seen. Importantly, there was no significant difference in disease progression or severity between the *CPA1 N256K* and *CPA1 N256K* × *Ddit3-KO* mice.

### Macrophage infiltration, acinar-to-ductal metaplasia, and fibrosis in *CPA1 N256K* × *Ddit3-KO* mice

**Figure 6:**
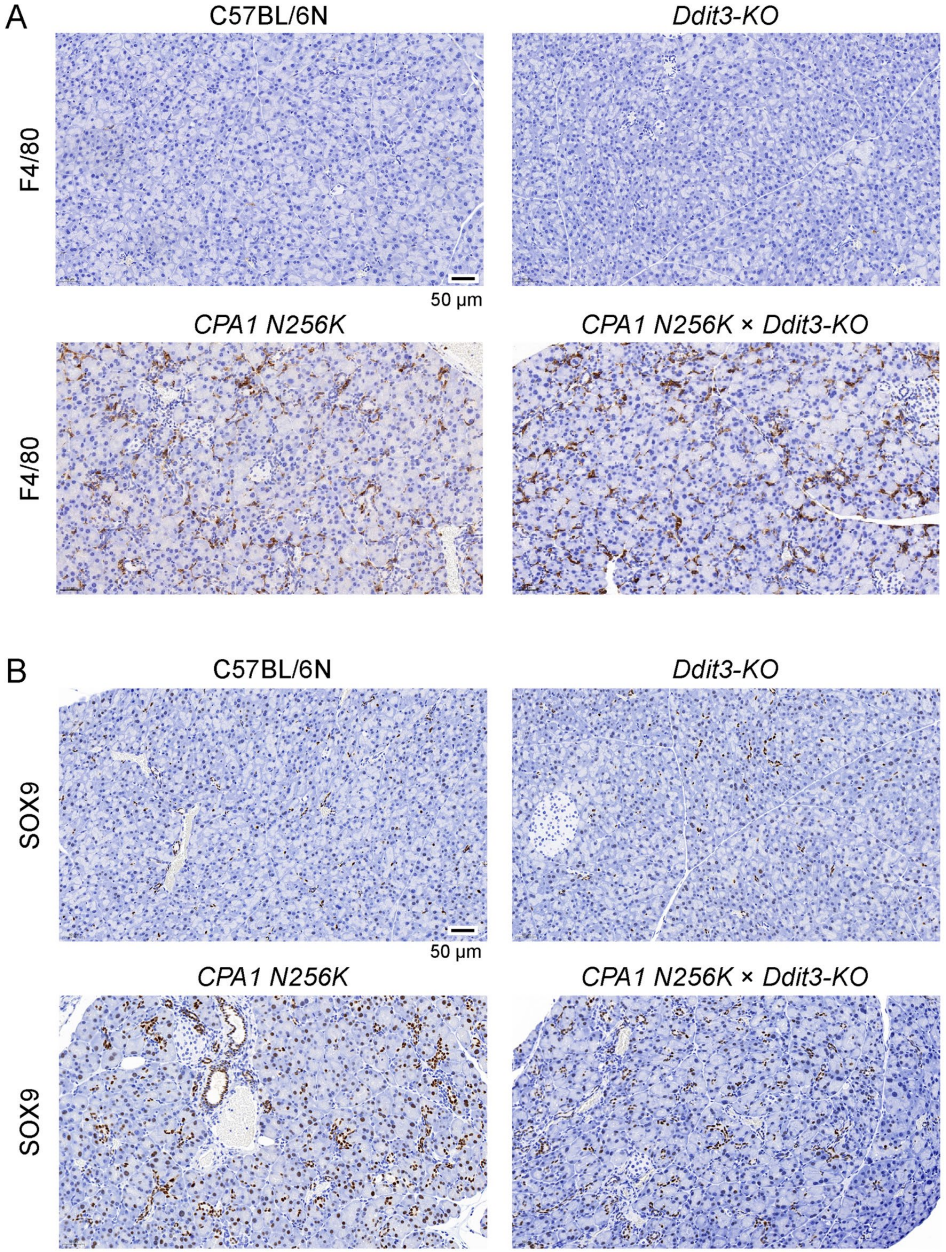
Macrophage infiltration and acinar-to-ductal metaplasia in the pancreas of *CPA1 N256K* × *Ddit3-KO* mice. (**A**) Immunohistochemistry staining of pancreas sections from 3-month-old C57BL/6N, *Ddit3-KO*, *CPA1 N256K,* and *CPA1 N256K* × *Ddit3-KO* mice for the F4/80 macrophage marker. Macrophages are stained brown. (**B**) Immunohistochemistry staining of pancreas sections from 3-month-old C57BL/6N, *Ddit3-KO*, *CPA1 N256K,* and *CPA1 N256K* × *Ddit3-KO* mice for the SOX9 ductal cell marker. Normal ductal cells and acinar cells that underwent ductal metaplasia are stained brown. The scale bar corresponds to 50 µm.

**Figure 7:**
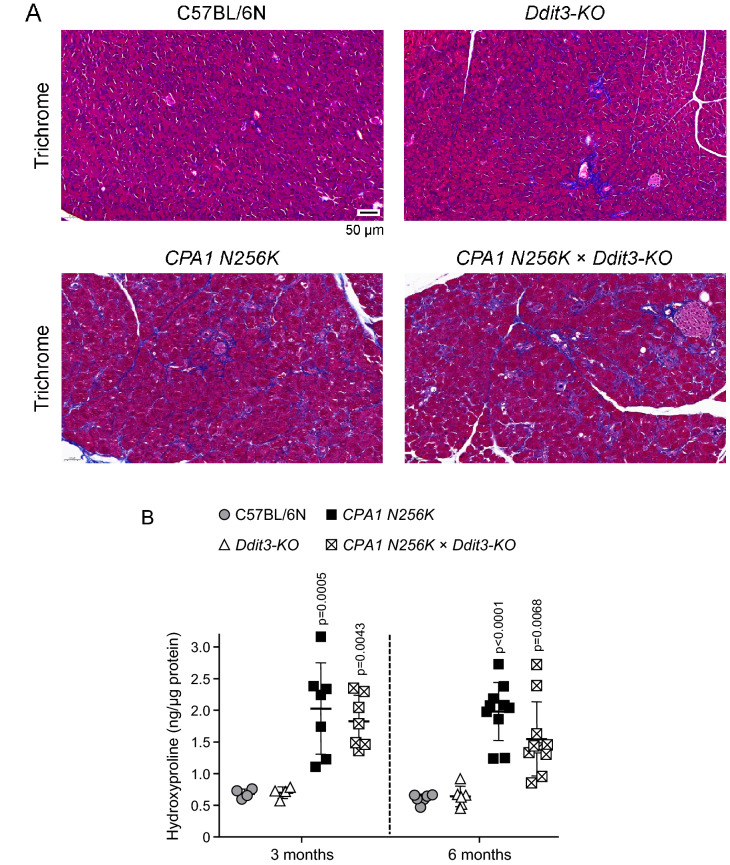
Fibrosis in the pancreas of *CPA1 N256K* × *Ddit3-KO* mice. (**A**) Masson’s trichrome staining of pancreas sections from 3-month-old C57BL/6N, *Ddit3-KO, CPA1 N256K,* and *CPA1 N256K* × *Ddit3-KO* mice. Collagen is stained blue. The scale bar corresponds to 50 µm. (**B**) Hydroxyproline content of the pancreas from C57BL/6N (n = 4 and 6), *Ddit3-KO* (n = 4 and 6), *CPA1 N256K* (n = 7 and 10), and *CPA1 N256K* × *Ddit3-KO* (n = 6 and 10) mice at 3 months and 6 months of age. Individual data points with mean (horizontal bar) and standard deviation are shown.

To visualize macrophage infiltration, pancreas sections were stained for the F4/80 marker using IHC (Fig. 6A). Widespread positivity was detected in pancreas sections of 3-month-old *CPA1 N256K* and *CPA1 N256K* × *Ddit3-KO* mice, with comparable intensity and distribution. Pseudotubular complexes due to acinar-to-ductal metaplasia are hallmark signs of chronic pancreatitis. IHC staining for the ductal cell marker SOX9 revealed widespread positivity in pancreas sections from 3-month-old *CPA1 N256K* and *CPA1 N256K* × *Ddit3-KO* mice, while the pancreas of C57BL/6N and *Ddit3-KO* mice showed only scattered staining of normal ducts (Fig. 6B). Finally, fibrosis was first investigated using Masson’s trichrome staining, which indicated the presence of diffuse fibrosis in 3-month-old *CPA1 N256K* and *CPA1 N256K* × *Ddit3-KO* mice but not in C57BL/6N and *Ddit3-KO* mice (Fig. 7A). Measurement of pancreatic hydroxyproline content confirmed the increased collagen levels in the pancreas of 3-month-old as well as 6-month-old *CPA1 N256K* and *CPA1 N256K* x *Ddit3-KO* mice relative to C57BL/6N and *Ddit3-KO* mice (Fig. 7B). We also performed IHC for alpha-SMA, however, only weak and scattered positivity were observed in the pancreas of *CPA1 N256K* and *CPA1 N256K* × *Ddit3-KO* mice, indicating a moderate extent of stellate cell activation (not shown). Taken together, the observations indicate similar severity of chronic pancreatitis in *CPA1 N256K* and *CPA1 N256K* × *Ddit3-KO* mice with respect to macrophage infiltration, acinar-to-ductal metaplasia, and fibrosis.

## Discussion

In this study, we demonstrate that the transcription factor DDIT3/CHOP plays no significant role in the onset, progression and severity of chronic pancreatitis in *CPA1 N256K* mice harboring a misfolding CPA1 variant. Since the *Ddit3/Chop* mRNA is significantly upregulated in the pancreas of *CPA1 N256K* mice and DDIT3/CHOP is known to mediate ER-stress associated apoptosis; it seemed reasonable to assume that pancreatic acinar cell death might occur through this mechanism. Therefore, the negative results of our experiments are surprising. We found that *CPA1 N256K* mice with a global deletion of *Ddit3/Chop* developed chronic pancreatitis with essentially identical features and timeline as the *CPA1 N256K* parent strain. Comparative evaluation of pancreas atrophy, plasma amylase elevations, histological damage, acinar-to-ductal metaplasia, macrophage infiltration, and fibrosis indicated no discernible differences. As expected, C57BL/6N and *Ddit3-KO* mice did not develop chronic pancreatitis and showed none of the morphological and biochemical changes described above.

The mechanistic role of DDIT3/CHOP in chronic pancreatitis has not been investigated before, although persistent activation in cerulein-induced disease was documented^31^. There are two published studies on acute pancreatitis in *Ddit3/Chop*-deleted mice^32,33^. The papers report somewhat more severe disease in the knockout strain, indicating a possible protective role of DDIT3/CHOP in acute pancreatitis. The mechanism of protection may be related to induction of apoptosis, which has been shown to mitigate inflammation by preventing pro-inflammatory necrosis^34^.

Transcription factor DDIT3/CHOP is ubiquitously expressed at relatively low levels and becomes significantly upregulated as a result of cellular stress, such as ER stress, nutrient deprivation, DNA damage, hypoxia or growth arrest (reviewed in^27^). Although it can affect a multitude of cellular functions, its best characterized role is the mediation of apoptotic cell death associated with chronic, unresolved ER stress^35–37^. DDIT3/CHOP upregulation is primarily driven by the PERK-eIF2α-ATF4 signal transduction pathway, however, the ATF6 and IRE1 pathways also contribute^27^. Activated PERK phosphorylates eIF2α, which results in reduced protein translation but paradoxically promotes translation of ATF4, which binds to the *DDIT3/CHOP* promoter in the nucleus. DDIT3/CHOP promotes apoptosis by altering the expression, directly or indirectly, of a variety of pro-apoptotic and anti-apoptotic genes (reviewed in^27,36,37^). The most pronounced effect, however, is the stimulation of protein synthesis, in concert with ATF4, which results in oxidative stress and cell death^38,39^.

At the onset of these studies, we assumed that acinar cell death would play a key role in the fibro-inflammatory process and protection against acinar cell death by deletion of *Ddit3/Chop* would prevent the development and/or progression of chronic pancreatitis. This assumption was also reinforced by the marked protective effect of *Ddit3/Chop* deletion against ER stress, β-cell apoptosis and diabetes in mouse models^40–42^. Clearly, this is not the case in the exocrine pancreas. One explanation might be that acinar cell death is not a requirement for disease initiation. Rather, stressed acini might secrete pro-inflammatory factors that attract macrophages and activate stellate cells. Extracellular ATP released by damaged acinar cells may mediate these events through calcium signaling^43^. Deletion of *Ddit3/Chop* has been shown to protect against fibrosis of various major organs such as the kidneys, lungs, liver, and heart (reviewed in^27,44^). In the pancreas, DDIT3/CHOP was implicated in protecting stellate cells from metabolic stressors and thereby promoting fibrosis^45^. We were unable to confirm the proposed pro-fibrotic role of DDIT3/CHOP, as Masson’s trichrome staining of pancreas sections and measurement of pancreatic hydroxyproline content revealed no significant difference in the extent of fibrosis between the *CPA1 N256K* and *CPA1 N256K* × *Ddit3-KO* mice. Deletion of *Ddit3/Chop* has been also associated with reduced macrophage infiltration during the inflammatory diseases of various major organs^27^. An interesting exception is the experimental model of liver fibrosis induced by dietary steatohepatitis. In this case, fibrosis was increased in *Ddit3/Chop*-deleted mice, likely due to a defect in CHOP-induced apoptosis of activated macrophages^46^. In our experiments, using semi-quantitative immunohistochemistry staining of pancreas sections for the macrophage marker F4/80, we did not observe a significant difference in macrophage infiltration between the *CPA1 N256K* × *Ddit3-KO* mice versus the *CPA1 N256K* parent strain.

In summary, our study conclusively demonstrates that in the *CPA1 N256K* mice, onset and progression of chronic pancreatitis is not dependent on the upregulation of *Ddit3/Chop*. The findings raise the possibility that the mild ER stress observed in the pancreas of these mice may not be pathogenic and CPA1 misfolding causes pancreatitis via other mechanisms. Alternatively, other important aspects of ER stress, not investigated here, such as ATP depletion and calcium overload, might drive pathology^47^. Limitations of our study include the global nature of *Ddit3/Chop* deletion and reliance on morphological rather than molecular analysis. Despite these potential shortcomings, the conclusions of the study are straightforward and rule out DDIT3/CHOP as a potential therapeutic target in misfolding-induced chronic pancreatitis.

## Supplementary Information

Supplementary Figure 1.

## Data Availability

Materials, data and protocols associated with this paper are available upon request.
