# Supplementary material for: Misfolding-induced chronic pancreatitis in CPA1 N256K mutant mice is unaffected by global deletion of Ddit3/Chop
Source: Sci Rep. 2022 Apr 15;12:6357. doi: 10.1038/s41598-022-09595-x (PMC9012826; doi:10.1038/s41598-022-09595-x)
Supplement: Supplementary file 1 — Supplementary Figure 1. [file 41598_2022_9595_MOESM1_ESM.pdf]

SUPPLEMENTARY MATERIAL

Misfolding-induced chronic pancreatitis in *CPA1 N256K* mutant mice is unaffected by global deletion of *Ddit3/Chop*

Balázs Csaba Németh[1#], Alexandra Demcsák[1#], Andrea Geisz[2], Miklós Sahin-Tóth[1]

[1]Department of Surgery, University of California Los Angeles, Los Angeles, California 90095; [2]Department of Molecular and Cell Biology, Boston University Henry M. Goldman School of Dental Medicine, Boston, MA 02118

Uncropped version of gels shown in Figure 1A

Figure 1A upper panel

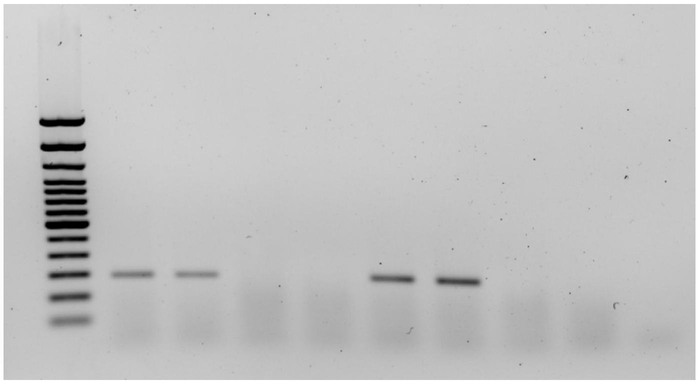

Figure 1A lower panel

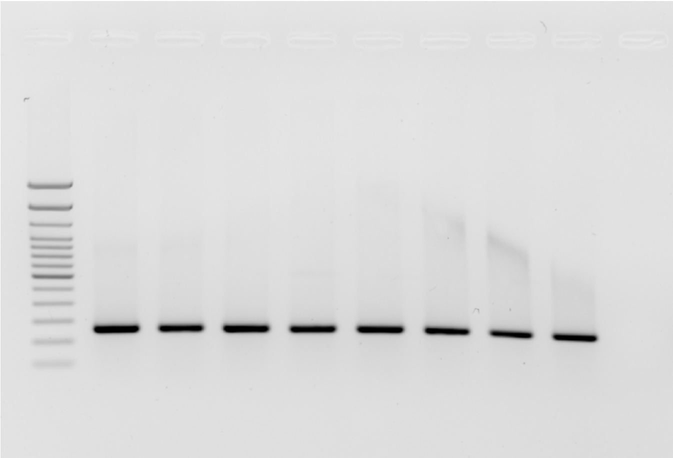

Supplementary Figure 1
